# Use of Ketamine Infusions for Treatment of Complex Regional Pain Syndrome: A Systematic Review

**DOI:** 10.7759/cureus.18910

**Published:** 2021-10-19

**Authors:** Ahish Chitneni, Anand Patil, Suhani Dalal, Joe H Ghorayeb, Yolanda N Pham, Gregory Grigoropoulos

**Affiliations:** 1 Family Medicine, Peconic Bay Medical Center/Northwell Health, Riverhead, USA; 2 Physical Medicine & Rehabilitation, New York-Presbyterian Hospital, New York City, USA; 3 Internal Medicine, Touro University California, Vallejo, USA; 4 Internal Medicine, Loma Linda University Medical Center, Loma Linda, USA; 5 Physical Medicine & Rehabilitation, University of Medicine and Health Sciences, New York City, USA; 6 Internal Medicine, Loyola University Chicago Stritch School of Medicine, Maywood, USA

**Keywords:** complex regional pain syndrome (crps), pain syndrome, infusion, chronic pain, ketamine

## Abstract

This systematic review aims to review clinical studies on the use of ketamine infusion for patients with treatment-resistant complex regional pain syndrome (CRPS).

The following systematic review was registered on the International Prospective Register of Systematic Reviews (PROSPERO) (CRD42021228470). Studies for the systematic review were identified through three databases: PubMed, Cumulative Index of Nursing and Allied Health Literature (CINAHL), and Cochrane Reviews. Inclusion criteria for studies consisted of randomized clinical trials or cohort studies that conducted trials on the use of ketamine infusion for pain relief in patients with CRPS. Exclusion criteria for studies included any studies that were systematic reviews, meta-analyses, case reports, literature reviews, or animal studies. In the included studies, the primary outcome of interest was the post-drug administration pain score.

In this systematic review, 14 studies met the inclusion criteria and were reviewed. In these studies, the dosage of ketamine infusion used ranged from 0.15 mg/kg to 7 mg/kg with the primary indication being the treatment of CRPS. In 13 of the studies, ketamine infusion resulted in a decrease in pain scores and relief of symptoms.

Patients who received ketamine infusion for treatment-resistant CRPS self-reported adequate pain relief with treatment. This suggests that ketamine infusion may be a useful form of treatment for patients with no significant pain relief with other conservative measures. Future large-scale studies, including randomized double-blind placebo-controlled trials on the use of ketamine infusion for CRPS, must be conducted in a large-scale population to further assess the effectiveness of ketamine infusion in these populations.

## Introduction and background

Complex regional pain syndrome (CRPS) presents with pain in an extremity or extremities following trauma. The typical patient is a middle-aged female with an upper limb insult, causing lasting pain and/or concurrent dysfunction in the sensory, motor, autonomic, and trophic domains [1]. CRPS is further classified into types I and II, the latter involving the identification of a specific nerve lesion. CRPS makes up approximately 1.2% of the diagnoses of chronic pain within the American patient population and 7% of patients post-trauma [2]. From a financial perspective, CRPS patients, on average, spent more on pain medications after their diagnosis. Due to its ill-defined characteristics, CRPS often requires management by a multidisciplinary team, thus incurring more expenses than the treatment of other chronic pain diagnoses. Compared to baseline before diagnosis, patients have a 2.17-fold increase in prescription costs and a 2.56-fold increase in total cost annually ranging from $3,888 to $4,845 [3]. 

Among the diverse therapies available for CRPS, including immunomodulation, hyperbaric oxygen therapy, and psychological therapy, medicinal approaches are typically the first-line therapy [1]. In particular, the open-channel N-methyl-D-aspartic acid or N-methyl-D-aspartate (NMDA) blocker ketamine is a medicinal interventional that has recently been studied. Ketamine is a phencyclidine or phenylcyclohexyl piperidine (PCP) derivative that initially became commercially available for human use in 1970 as a rapid-acting intravenous (IV) anesthetic. It is currently classified by the Food and Drug Administration (FDA) as an anesthetic induction agent in doses ranging from 1 to 4.5 mg/kg [4]. Ketamine has proven to be a desirable drug, despite its induction of dissociative effects and abuse potential [5-6]. It is favorable due to its short half-life and lack of clinically significant respiratory depression [7]. In addition to its anesthetic effects, ketamine possesses analgesic, anti-inflammatory, and antidepressant activities. These characteristics bode well for the treatment of CRPS for which treatment is approached through a biopsychosocial model of chronic pain and an interdisciplinary approach to the management of CRPS [8]. 

Pharmacology and mechanism of action of ketamine

Ketamine is composed of a chiral center at the C-2 carbon of the cyclohexanone ring so that two enantiomers exist: S(+)-ketamine and R(-)-ketamine. The S-enantiomer has greater pharmacological potency due to a greater affinity for the PCP-binding site on the NMDA. Accordingly, preparations containing only the S-enantiomer are desirable.

Traditionally, ketamine is administered via IV or intramuscular routes. Alternative routes of administration include insufflation/intranasal, inhalational, oral, topical, epidural, and rectal modes. It is both water and lipid-soluble. These qualities allow for its rapid distribution throughout the body and ability to cross the blood-brain barrier. The predominant route of metabolism is via hepatic cytochrome P450 enzymes, with 12% remaining protein-bound in plasma. Ketamine's half-life in plasma is approximately 2.5 to 3 hours, rapidly metabolizing to norketamine, hydroxynorketamine, and dehydronorketamine. Approximately 4% of the unmetabolized drug is excreted via the urine [9].

The therapeutic range of ketamine makes it one of the safest sedative agents for most emergency clinical and preclinical situations [10]. In low doses, it causes analgesia and sedation, and in high doses, it produces general anesthesia. Administration of ketamine typically increases heart rate, systolic and diastolic blood pressure, salivary and tracheobronchial secretions, and bronchodilation [11]. It has minimal effects on airway reflexes and respiratory rate. Currently, three pain societies recommend intravenous dosing of ketamine for chronic pain at 0.5 to 2 mg/kg for one-day outpatient or three- to five-day inpatient treatment with higher doses titrated to effect [10].

Ketamine exerts its effects through a variety of pathways. Its primary mechanism is as a non-competitive antagonist at the PCP-binding site of the NMDA receptors in the central nervous system (CNS) [12]. At this site, it decreases the frequency of channel opening and duration of time spent in the active, open state [13]. The NMDA receptor is a ligand-gated channel whose major endogenous agonist is glutamate, the predominant excitatory neurotransmitter in the CNS. Inhibition of the NMDA receptor results in decreased neuronal activity.

Ketamine also acts on other non-NMDA pathways that affect pain and mood regulation. It acts as an antagonist of nicotinic and muscarinic cholinergic receptors, as well as blocking sodium and potassium channels. Ketamine activates high-affinity D2 dopamine receptors and l-type voltage-gated calcium channels, promotes γ-aminobutyric acid A (GABA-A) signaling, and enhances descending modulatory pathways [14-15].

Given the high co-prevalence rate of chronic pain and depression, the antidepressant and post-traumatic stress disorder (PTSD) relieving effects of ketamine have generated interest in the psychiatric community. The mood-enhancing effects of ketamine have been shown to emerge about four hours after intravenous administration, well after the drug has been cleared from the bloodstream. This suggests a neuromodulatory effect that persists for one to two weeks [16].

This article was previously posted to the medRxiv preprint server on April 27, 2021.

## Review

Methods

The following systematic review was registered on the International Prospective Register of Systematic Reviews (PROSPERO) website (CRD42021228470) and the systematic review follows the guidelines listed by the Preferred Reporting Items for Systematic Reviews and Meta-Analyses (PRISMA) statement. In the review, studies describing the use of ketamine infusion for CRPS were researched to validate the use of the treatment.

Search Strategy

An electronic literature search using three databases was conducted by the team. The databases PubMed, Cochrane Reviews, and Cumulative Index of Nursing and Allied Health Literature (CINAHL) were searched using the terms “Ketamine” or “Ketamine infusion” and “CRPS” or “Complex Regional Pain Syndrome” to find peer-reviewed clinical studies. No date limiters were used. The initial search resulted in 101 non-duplicate entries from the three databases. After the application of inclusion/exclusion criteria, 87 articles were excluded from the search and 14 studies were included in the study.

Inclusion and Exclusion Criteria

Inclusion criteria consisted of all studies that were randomized controlled trials or studies that observed the effect of the use of ketamine infusion for pain relief in patients with CRPS. Exclusion criteria consisted of nonpeer-reviewed studies, systematic or meta-analysis reviews, case reports, and animal studies. All articles were reviewed by both reviewer AP and reviewer AC separately and any disagreements were resolved by tiebreaker reviewer SD. After the review process, both reviewer AP and AC had a 95% agreement rate for the studies to be included.

Data Extraction

Of the studies to be included in the paper, the following data were extracted for discussion: 1) length of ketamine infusion, 2) follow-up period, 3) dose of ketamine used, 4) outcomes of interest, and 5) post-procedural pain scores.

Results

Study Characteristics

**Figure 1:**
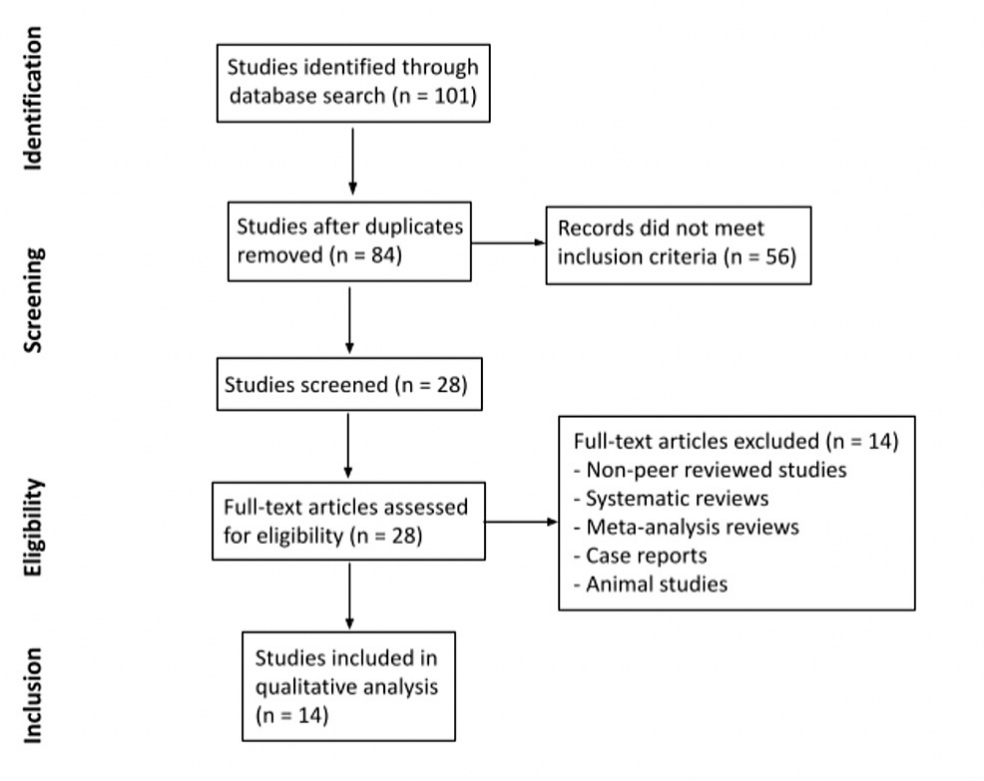
Flow Chart of the Study Selection and Inclusion Process

A total of 101 articles were identified as meeting the keyword search between the databases. After removing duplicates, 84 articles were screened, and 56 did not meet inclusion criteria based on their respective titles and abstracts. The remaining 28 articles were reviewed in full text and an additional 14 articles were excluded due to meeting exclusion criteria. Fourteen total articles were selected to be included in this review (Figure 1). A total of 455 patients were analyzed between the 14 included studies. Sample sizes ranged from four to 114 human subjects. Participant ages ranged from 12 to 68 years. Follow-up periods ranged from three hours to five years, with four studies having a follow-up of less than one week (three hours to five days), eight studies with one to six months, and two studies from three to five years.

All studies used intravenous ketamine infusions as the intervention. Five of the 14 studies used escalating, titrated doses, while the other nine studies used the same dosage throughout the duration of the intervention. Duration of analgesia ranged from five minutes to 10 days based on the dosage of ketamine used and time length measured in each study. Most studies tailored ketamine dosages based on individual weight, with doses ranging from 0.15 to 7 mg/kg/hour. Kirkpatrick et al., Kiefer et al., and Goldberg et al. did not calculate dosages based on patient weight and instead used standardized dosages between 10 to 200 mg/hour [17-19].

All studies but one used some form of subjective pain scales, such as the visual analog scale (VAS) or verbal numeric rating scale (NRS), which range from 0 (no pain) to 10 (worst possible pain). Instead of a pain score, Kirkpatrick et al. measured pain threshold, which is defined as the amount of stimulation before the pain is experienced [17]. In 13 of the studies, ketamine infusion resulted in a decrease in pain scores and threshold. Kiefer et al. did not find a significant change in pain relief in their sample of patients with longstanding, severe CRPS [18]. They posit this may be due to non-NMDA-receptor-mediated mechanisms that exist in patients with severe CRPS refractory to numerous treatments, essentially rendering ketamine non-effective.

Other measured outcomes included morphine-equivalent intake, plasma levels of ketamine, quality of life, functional improvement, cognitive effects, and side effects of ketamine infusion. Sheehy et al. measured oral morphine-equivalent opioid intake and found that while ketamine reduced pain intensity, it did not reduce overall opioid intake [20]. Goldberg et al. also measured plasma levels of ketamine and found that pain relief correlated with maximum plasma levels of ketamine and norketamine, indicating that downstream metabolites may play a role in therapeutic effect [19]. Kiefer et al. and Schwartzman et al. also measured quality of life (QOL) as an outcome [21-22]. Data from Kiefer et al. supported a significant improvement in QOL in the majority of patients at three and six months following treatment, while Schwartzman et al. found that there were no significant changes in QOL scores following treatment in either the treatment or placebo groups [21-22].

Functional improvement, including the ability to work, activity level, active range of motion, and effect on movement disorder, was measured in four studies. Kiefer et al. showed that ability to work was significantly improved three months post-treatment, with an even greater improvement by six months [21]. Schwartzman et al. found that based on activity watch data, there were no significant changes in activity level between pre- and post-treatment, but the number of nighttime awakenings in the ketamine group decreased by 85% post-treatment [22]. Puchalski et al. and Sigtermans et al. did not show that treatment caused functional improvement in active range of motion and the ability to walk without support [23-24].

Cognitive effects were assessed by Koffler et al. who found that there were significant improvements in brief auditory attention and processing speed, no changes in learning, memory, nor motor speed, and a slight decline in motor strength [25]. Side effects of the ketamine infusion were directly assessed in seven of the 14 studies. The majority of studies reported mild general symptoms of fatigue, headache, and nausea. Several studies also reported participants with side effects of vomiting, feeling of inebriation, and sedation. Two studies by Correll et al. and Sigtermans et al. reported patients with mild-to-moderate psychotomimetic effects, such as hallucinations [26-27].

The findings from all 14 included studies are summarized in Table 1. Overall, a majority of the 14 studies produced positive results for the effect of ketamine infusions on improved pain control and function for the treatment of CRPS. This was achieved by improved VAS or NRS pain scores or pain threshold. Increased quality of life and positive changes in physical and cognitive function in CRPS patients treated with ketamine were also reported. One study did not show a significant change in pain relief with ketamine infusion in patients with severe disease [18].

**Table 1:** Summary of Findings From Included Studies AROM: active range of motion; CBC: complete blood count; CRPS-1: complex regional pain syndrome type 1; h: hour; IV: intravenous; LFTs: liver function tests; QOL: quality of life; QST: quantitative sensory testing; RCT: randomized controlled trial; RK: racemic ketamine; ROM: range of motion; S+K: S+ketamine; VAS: visual analog scale

Author	Year	Study	Sample Size	Average Age (Range) (years)	Follow-up Period	Intervention	Ketamine Dose Used	Measures	Conclusions
Sheehy et al. [20]	2015	Longitudinal, cohort	63	15 (12 - 17)	1 month	Subanesthetic ketamine infusions - 4 - 8 h per day for 3 days	0.1 - 0.3 mg/kg/h	Self-reported pain scores, morphine-equivalent intake, side effects	Ketamine significantly reduced pain intensity and yielded greater pain reduction but did not change overall morphine-equivalent intake. Ketamine was not associated with psychotropic effects at doses given.
Schwartzman et al. [22]	2009	Randomized control trial	19	42 (24 - 60)	3 months	IV ketamine infusions 4 h (25 ml/h) daily for 10 days	0.35 mg/kg/h	Pain questionnaires, activity watch data, quantitative sensory testing, quality of life (QOL), side effects	IV ketamine administered in an outpatient setting resulted in significant reductions in many pain parameters, while the placebo group demonstrated no treatment effect in any parameter. These results warrant a larger RCT using higher doses of ketamine and a longer follow-up period.
Sigtermans et al. [24]	2009	Randomized control trial	48 females	45 (33 - 57)	3 months	Individually tailored IV low- dose ketamine infusions held for 4.2 days	22.2 ± 2.0 mg/h/70 kg (0.07 mg/kg/hr - 0.43 mg/kg/hr)	Pain score, skills questionnaire, AROM, touch sensitivity, skin temperature, volumetric measurement, LFTs, side effects	In a population of mostly chronic CRPS-1 patients with severe pain at baseline, a multiple-day ketamine infusion resulted in significant pain relief without functional improvement. Treatment with ketamine was safe with psychotomimetic side effects that were acceptable to most patients.
Puchalski et al. [23]	2016	Observational study	5 females	34 (25 - 45)	1.5 months	4-hour infusion of anesthetic IV ketamine dosage over 10 days	1.5 mg/kg/h	Pain score, presence of allodynia, ROM, ability to walk without support	This beneficial analgesic effect was confined to 1.5 - 2.5 months after treatment and then pain relapsed to the baseline level. These results show a short-term analgesic effect for this therapy, with no effect on movement and function of the affected limbs.
Sigtermans et al. [27]	2010	Observational study	10	43.2 ± 13.0	3 hours	Seven IV 5-minute long low-dose ketamine infusions with increasing doses at 20 min intervals	.02 mg/kg - .15 mg/kg	Spontaneous pain ratings, VAS response to experimental heat stimuli	While ketamine's effect on acute experimental pain was driven by pharmacokinetics, its effect on CRPS pain persisted beyond the infusion period when drug concentrations were below the analgesia threshold for acute pain. This indicates a disease modulatory role for ketamine in CRPS-1 pain, possibly via desensitization of NMDA receptors in the spinal cord or restoration of inhibitory sensory control in the brain.
Correll et al. [26]	2004	Retrospective case notes analysis	33	40 (20 - 68)	3 years	Continuous subanesthetic IV ketamine infusion for 4.7 days	23.4 mg/kg	Pain scale, degree of relief, side effects	Limited subanesthetic inpatient infusions of ketamine may offer a promising therapeutic option in the treatment of appropriately selected patients with intractable CRPS. Further studies are needed to establish the safety and efficacy of this novel approach.
Kirkpatrick et al. [17]	2020	Observational study	114 (89 females and 25 males)	39 (9 - 50)	3 days	Escalating dose ketamine IV infusion	Start at 60 mg/hr, titrated up to 200 mg/hr on Day 4	Pain threshold	Four days of treatment are sufficient for the treatment of CRPS of the lower extremities. For the upper extremities, > 4 days may be required. This was the first study to utilize quantitative sensory testing to direct the treatment of a chronic pain disorder.
Goebel et al. [28]	2015	Observational study	5	44 (37 - 55)	6 months	Low-dose, 4.5-day RK infusion	.9 mg/kg/hr	Pain score, side effects, LFT/CBC	IV infusion of subanaesthetic doses of S+K over 4.5 days can substantially reduce pain in longstanding CRPS for several weeks.
Dahan et al. [29]	2010	Randomized control trial	60	46 ± 12	2.5 months	100-h titrated ketamine or placebo infusion	0.07 mg/kg/hr – 0.43 mg/kg/hr	Pain scores, blood samples	Long-term S(+)-ketamine treatment is effective in causing pain relief in CRPS-1 patients with analgesia outlasting the treatment period by 50 days. These data suggest that ketamine initiated a cascade of events, including desensitization of excitatory receptor systems in the central nervous system, which persisted but slowly abated when ketamine molecules were no longer present.
Patil et al. [30]	2012	Retrospective case notes analysis	49	45 (18 - 68)	5 years	IV ketamine infusions, pretreatment with midazolam and ondansetron	0.5 mg/kg given over 30 - 45 minutes	Pain score, long-term pain relief, previous interventions, side effects	In patients with severe refractory pain of multiple etiologies, subanesthetic ketamine infusions may improve VAS scores. In half the patients, the relief lasted for up to 3 weeks with minimal side effects.
Kiefer et al. [18]	2008	Open label trial	20	30.4 (14 - 48)	6 months	5-day anesthetic ketamine dosage	7 mg/kg/h	Pain score, effect on movement disorder, QOL, ability to work	Anesthetic ketamine in previously refractory CRPS patients leads to benefits in pain reduction, associated CRPS symptoms, improved quality of life, and ability to work. An RCT will be necessary to prove its efficacy.
Koffler et al. [25]	2007	Observational study	9	29 (19 - 41)	1.5 months	5 days of anesthetic ketamine infusion	3 - 7 mg/kg/h	Acute and overall pain, brief attention and processing speed, cognitive domains, motor strength	Deep ketamine therapy is effective for relief of pain CRPS-I. There were no adverse cognitive effects of extended treatment with deep ketamine infusion. No definitive conclusions could be drawn about the relationship between mood and personality factors and the presence of CRPS-I.
Kiefer et al. [21]	2008	Clinical trial	4	25 (18 - 43)	2 days	S(+)-ketamine infusions, titrated over a 10-day period	50 mg/day - 500 mg/day	VAS pain, thermo- and mechanical detection thresholds, side effects	S(+)-ketamine can be gradually titrated to large doses (500 mg/day) without clinically relevant side effects. There was no pain relief or change in QST measurements in this series of longstanding severe CRPS patients.
Goldberg et al. [19]	2010	Clinical trial	16	33 (17 - 47)	5 days	Titrated ketamine infusion maintained for 5 days	10 - 40 mg/hour	Pain scale, plasma levels of ketamine	The pain relief experienced on Day 2 of the infusion continued to improve over the 5-day infusion period and correlated with the maximum plasma levels of ketamine and norketamine. Downstream metabolites of ketamine and norketamine might be playing a role in its therapeutic efficacy.

Discussion

A double-blind placebo-controlled study observing the effects of outpatient IV ketamine for the treatment of CRPS was conducted by Schwartzman et al. [22]. In this study, all subjects diagnosed with CRPS with pain for more than six months and who failed conservative therapy, such as nerve blocks, opioid analgesics, and NSAIDs, were included in the trial. Subjects receiving ketamine treatment were infused with 25 ml/h daily for four hours for a 10-day treatment period. All subjects who received both ketamine and the placebo were given treatment with clonidine to suppress any side effects. Results showed that the IV ketamine group had a statistically significant reduction in their pain parameters, while the placebo group showed no treatment effect in the pain parameters. To evaluate pain, all subjects completed two pain questionnaires prior to treatment and one pain questionnaire every week for 12 weeks post-treatment. For this study, the Numerical Pain Rating Scale (NPRS) questionnaire was used which was scaled from 0 - 10. For the ketamine group, significant decreases in all parameters post-treatment were observed. Of note, the decreases in pain parameters lasted the entirety of the 12-week treatment course. Pain in the affected area, burning pain, allodynia, and overall pain level were all parameters that had significant decreases in the ketamine group. Given the decrease in pain scores in these specific parameters, the use of ketamine likely had a significant overall improvement in quality of life and may be considered as an option for patients who have failed conservative measures, such as the subjects in this trial.

Another study by Sigtermans et al. explored the use of ketamine on pain relief for patients with CRPS type 1 (CRPS-1). In the study, 60 CRPS-1 patients were included in a double-blind randomized placebo-controlled trial [24]. Notably, the included group in this study consisted of patients with a diagnosis of CRPS-1 referred to the clinic regardless of the previous history of response to conservative treatment. In the other studies discussed in this review, many patients included in a ketamine trial had failed conservative treatment for a minimum number of months prior to receiving ketamine infusion. Patients were given a four-day infusion of ketamine with a dose of 22 mg/h/70 kg. All subjects were evaluated based on pain scores over the 12-week study period. Results showed patients receiving ketamine had significantly lower pain scores than patients receiving placebo treatment. Although significance was established in the ketamine group, it is notable that by week 12, the significant inter-group difference in pain relief was lost. Patients had no functional improvement with the ketamine treatment and also experienced mild to moderate psychomimetic side effects.

A longitudinal study by Sheehy et al. observing the effects of subanesthetic ketamine infusions for the treatment of children and adolescents with chronic pain was also reviewed [20]. In this study, the effects of ketamine on both CRPS and other chronic pain syndromes were compared. Sixty-three children received IV administration of subanesthetic doses of ketamine in an outpatient setting with dosing ranging from 0.1 to 0.3 mg/kg/h that lasted for four to eight hours per day. Similar to the other studies, the primary outcome measure was a change in pain scores using a numeric rating scale. A primary result of the study was that the effect of ketamine on pain scores varied based on the subject's pain diagnosis. Overall, the use of ketamine resulted in the greatest pain score reductions in patients with CRPS than any other chronic pain condition. In addition, multivariate analysis was able to further qualify that CRPS was a significant predictor of higher pain score reductions after ketamine treatment. Although targeting pediatric patients, this study further provides validity that ketamine may be safely used for chronic pain syndromes, particularly CRPS.

The prolonged painful stimulus causes an increased release of glutamate from nociceptive afferents onto the dorsal horn neurons. This glutamate stimulates NMDA receptors on second-order neurons which leads to an increase in pain intensity (windup) and causes central sensitization. Correl et al. posit that blocking these NMDA receptors is what propagates ketamine’s ability to treat CRPS [26]. Temporally, results showed that a patient with 20 years of CRPS had full suppression of pain after the infusion of ketamine, pointing towards a neuromodulatory effect. The mean survival of pain relief was found to be three to six months with only one dose and up to one to three years with a second infusion. Interestingly, the ideal infusion rate was found to be 15 - 20 to 20 - 25 mg/hr over the course of 10 to 20 days, with those receiving more than 35 mg/hr categorized as non-responders or having exacerbated side effects. Side effects are a primary concern with ketamine therapy with potential neurotoxic effects observed in animal studies. It is yet to be seen how well these findings correlate in humans; therefore, the predominant approach is to dose at constant low rates. The most common side effect reported was feeling intoxicated, followed by hallucinations as seen in 18% of the study population. Although the results are highly encouraging, the study population contained a large number of early CRPS diagnoses with no established protocol for treatment and assessment of results.

At the pharmacological level, Sigtermans et al. studied the S(+) variant of the compound due to its greater availability and two-fold greater analgesic potency than S(-) or racemic mixtures [27]. They found a plasma concentration-driven effect on acute pain which showed an “on-off” phenomenon that inhibited NMDR in the central pain circuit. The study tested 10 females and administered seven IV infusions that were five minutes long of low-dose ketamine with increasing doses at 20 min intervals. Of note, the study found it difficult to blindly administer S(+) ketamine and compare it to placebo due to two main reasons. One, the effects of ketamine on patient affect were apparent to the administrator. Second, creating a placebo with similar behavioral effects (i.e., drowsiness) was difficult due to the mixing of drugs that might have independent pain modification processes. It is advocated that a de-chronification of pain occurs secondary to long-term desensitization of upregulated NMDA receptors. Further research is required to differentiate this mechanism of action versus top-down inhibitory control of pain sensory systems.

Escalating doses of ketamine infusion, starting at 60 mg/hr to 200 mg/hr, based on quantitative sensory testing to direct treatment, demonstrate that ketamine has its most potent effect on regions of pain. A distinction is made when treating extremity pain in patients with CRPS, with pain threshold plateaus being realized between three and four days, and greater than four days of ketamine treatment for patients with CRPS of the lower and upper extremities, respectively [27]. Similarly, long-term (100-h) escalating doses of ketamine infusion starting at 5 mg/hr/70 kg to 20 mg/hr/70 kg demonstrate effectiveness in pain relief for CRPS-1 patients, with analgesia outlasting the treatment period by 50 days. This observation suggests that ketamine initiates a cascade of events, including desensitization of excitatory receptor systems in the CNS which persist but abate slowly, even when ketamine molecules are no longer detectable in the body.

A five-year retrospective analysis by Patil and Anitescu studying the efficacy of outpatient ketamine infusions revealed that 50% of patients with severe refractory pain of multiple etiologies realized pain relief for up to three weeks with minimal side effects after receiving mean ketamine doses per infusion of 0.9 (± 0.4) mg/kg with a median duration between infusions of 233 days [30]. All patients were pretreated with midazolam, ondansetron, and an initial ketamine dose of 0.5 mg/kg given over 30 to 45 minutes. If the initial dose was effective, it was continued in subsequent infusions. They were then scheduled to receive treatment dosages every three to four weeks per pain clinic protocols. If well-tolerated, the dose was increased at subsequent infusions to the highest tolerated dose producing analgesia without unacceptable side effects. While these patients’ long-term results are unavailable per this protocol, Kiefer et al. noted long-term complete pain relief, six months post-treatment, in 50% of patients with advanced and refractory CRPS who underwent a five-day anesthetic ketamine infusion protocol [21]. Anesthesia was induced by bolus injection of ketamine (1 - 1.5 mg/kg) and midazolam (2.5 - 7.5 mg). Tracheal intubation was facilitated by vecuronium (0.1 mg/kg). Treatment was maintained by infusions of ketamine over five days, starting at 3 mg/kg/h, followed by a gradual daily titration up to a final dose of 7 mg/kg/h. Midazolam was co-administered and adjusted as required to obtain a stable level of deep sedation and to attenuate ketamine-specific agitation.

Ketamine infusions can provide significant analgesia to patients with CRPS that is refractory to conservative methods of pain management. One observational study by Goebel et al. evaluated the short- and long-term analgesic effects of 4.5-day, low-dose (0.9 mg/kg/hr) racemic ketamine (RK) infusions in five patients with refractory CRPS [28]. The study authors found that intravenous infusion of low, subanesthetic doses of RK over 4.5 days can substantially reduce pain in patients with CRPS with 60% of patients reporting substantial pain relief during the treatment period. The median pain intensity at the beginning of the treatment period was NRS 8.5 and was reduced to NRS 5 on the last treatment day. The authors concluded that low-dose RK infusion over the duration of 4.5 days provided beneficial analgesia for short durations, but further prospective trials were needed to understand long-term pain control with multiple repeat ketamine infusions. Koffler et al. conducted an observational study to understand pain control and neurocognitive effects of a five-day anesthetic ketamine infusion in patients with CRPS [25]. The study evaluated intellectual and academic abilities, executive functioning and processing speed, attention, learning, memory, and motor functioning in nine patients pre-treatment and six weeks post-treatment. The study population demonstrated significant pain reduction (present pain index (PPI); t(8) = 2.393, p = .044) without adverse cognitive effects of ketamine infusion at six weeks post-treatment.

Ketamine, norketamine, and the downstream metabolites likely play an important role in the analgesic properties and efficacy of ketamine infusions. Goldberg et al. conducted an observational study to determine the effects of a five-day moderate dose, continuous racemic ketamine infusion while evaluating for S-ketamine, R-ketamine, S-norketamine, and R-norketamine plasma levels [19]. Sixteen patients with CRPS enrolled in an observational cohort study were assessed daily for pain and plasma concentrations of ketamine and norketamine. Concentrations were measured before initiation of infusion, at several time points during the infusion, on Days 2 through 5 of the infusion, and 60 minutes after the conclusion of infusion on Day 5. The study authors found that the ketamine infusion resulted in significant pain reduction by the second day of infusion which correlated with peak plasma concentrations, supporting the antinociceptive properties of the drug. 

While several studies have shown ketamine infusions to be an effective analgesic for pain management in patients with refractory CRPS, a pilot open-label study by Kiefer et al. analyzing the efficacy of subanesthetic ketamine infusion in refractory CRPS patients found that treatment did not offer pain relief [18]. Four study patients with refractory CRPS were enrolled to receive continuous S+-ketamine infusions gradually titrated (50 mg/day - 500 mg/day) over a 10-day period. Pain intensity and side effects on 100-mm VAS during a four-day baseline, over 10 treatment days, and two days post-treatment were measured. The subjective pain relief with ketamine infusions was 4.6 ± 1.6 mm on the 100-mm VAS. Over the 10 treatment days, there was no significant difference in pain relief between the treatment and baseline pain scores.

Future Studies

Overall, the included studies generally reported adequate pain relief with varying protocols of ketamine infusion for treatment-resistant CRPS. Heterogeneity was significant in these studies due to variations in dosage, protocols, a number of subjects, combinations of pharmacological agents administered, and rescue analgesics. Despite these differences, some valuable trends can be observed and may provide direction in future studies for the utility of ketamine infusion. Of interest would be identifying the ideal time course in the treatment regimen to use ketamine for maximal efficacy. Also, dosage stratification should be explored based on age, the severity of pain as per numerical scale ratings, and time of living with the disease. With new innovations in pain management, such as neurostimulation and implantable pumps, a comparative study with ketamine should also be undertaken to understand the safest way to address pain control. Ketamine’s concurrent influence on psychology should be further understood as pain is a complex entity that affects numerous functionalities. In addition, the biopsychosocial model of pain and the interdisciplinary approach that can be taken to alleviate pain secondary to CRPS must be studied. In many cases, the use of ketamine has been shown to relieve symptoms of depression and PTSD so the effect of ketamine on pain relief, mental health benefits, and the social effects, such as improvement of quality of life, are all potential areas of study. 

Strengths and Limitations

The strengths of this review included select studies that were published in peer-reviewed journals with thorough methodology and low dropout rates. However, this systematic review also had several notable limitations, including significant heterogeneity of the included studies in regard to study design, sample size, dosing strategies, and tools used to measure variables and outcomes. Among the 14 studies reviewed (yielding a total of 455 included patients), several confounding factors were identified, including variable ketamine dosing strategies and durations of treatment, observational design in studies, and the use of other nonopioid multimodal analgesia. Study subjects were assessed for pain scores and other outcomes at varying intervals, which may influence outcomes of recall, in turn, increasing the risk of recall bias. Moreover, pain is subjective and challenging to measure despite the utilization of validated scales.

## Conclusions

Patients who received ketamine infusion for treatment-resistant CRPS reported adequate pain relief with treatment. Given the results, this suggests that ketamine infusion may be a useful form of treatment for patients with no significant pain relief with other conservative measures. Although pain scores were reduced with ketamine use in all but one study reviewed, parameters, such as functional improvement and quality of life, were not as well studied. In addition, one of the included studies observed the effects of ketamine in pediatric patients with chronic pain syndromes signaling a scope for use in younger patients. Future large-scale studies, such as randomized double-blind, placebo-controlled trials, must be conducted to better correlate the use of ketamine infusion in CRPS patients with improved pain scores, changes in parameters (such as quality of life and activities of daily living), and effectiveness by age cohorts.
